# Lower peripheral helper T cell levels in the synovium are associated with a better response to anti-TNF therapy in rheumatoid arthritis

**DOI:** 10.1186/s13075-020-02287-9

**Published:** 2020-08-25

**Authors:** Antonio Julià, Gabriela Ávila, Raquel Celis, Raimon Sanmartí, Julio Ramírez, Sara Marsal, Juan D. Cañete

**Affiliations:** 1 Rheumatology Research Group, Vall d’Hebron Research Institute, Vall Hebron University Hospital, Pg Vall Hebron 119-120, 08035 Barcelona, Spain; 2 Rheumatology Department, Hospital Clínic de Barcelona i IDIBAPS, Barcelona, Spain

**Keywords:** Rheumatoid arthritis, Anti-TNF therapy, Synovial membrane, Clinical response, Deconvolution, Peripheral T helper, Gene expression, Immunofluorescence

## Abstract

**Background:**

The mechanisms by which only some rheumatoid arthritis (RA) patients respond favorably to TNF blockade are still poorly characterized. The goal of this study was to identify biological features that explain this differential response using a multilevel transcriptome analysis of the synovial membrane.

**Methods:**

Synovial samples from 11 patients on anti-TNF therapy were obtained by arthroscopy at baseline and week 20. Analysis of the synovial transcriptome was performed at the gene, pathway, and cell-type levels. Newly characterized pathogenic cell types in RA, peripheral helper T cells (T_PH_), and CD34-THY1+ fibroblasts were estimated using a cell-type deconvolution approach. T_PH_ association was validated using immunofluorescence. External validation was performed on an independent dataset.

**Results:**

After multiple-test correction, 16 and 4 genes were differentially expressed at baseline and week 20, respectively. At the pathway level, 86 and 17 biological processes were significantly enriched at baseline and week 20, respectively. Longitudinal expression changes were associated with a drastic decrease of innate immune activity (*P* < 5e−30), and an activation of the bone and cartilage regeneration processes (*P* < 5e−10). Cell-type deconvolution revealed a significant association between low T_PH_ cells at baseline and a better response (*P* = 0.026). Lower T_PH_ cells were maintained in good responders up to week 20 (*P* = 0.032). Immunofluorescent analyses confirmed the accuracy of the cell-type estimation (*r*^2^ = 0.58, *P* = 0.005) and an association with response. T_PH_ association with anti-TNF response was validated in an independent sample of RA patients (*P* = 0.0040).

**Conclusions:**

A lower abundance in the synovial membrane of the pathogenic T cell type newly associated with RA, peripheral helper T lymphocyte, is associated with a good response to anti-TNF therapy. Major changes in the myeloid cell compartment were also observed in response to therapy. The results of this study could help develop more effective therapies aimed at treating the pathogenic mechanisms in RA that are currently not well targeted by anti-TNF agents.

## Background

Anti-TNF therapies have been a major breakthrough in the management of rheumatoid arthritis (RA) [1]. To date, disease remission has become an attainable goal for many patients [2]. However, 30 to 40% of anti-TNF-treated patients do not show a significant clinical improvement [3]. The reasons behind this differential response are still elusive [3]. Clinical and basic studies have attempted to identify key factors, with very limited success [4]. Identifying the determinants of anti-TNF response would be of high value: these could be used to stratify patients, guide drug selection, and improve disease management, and could help to develop more efficacious therapies, either alone or in combination with anti-TNF agents.

The synovial membrane is the target tissue of chronic inflammation in RA [5], and its analysis should provide highly valuable information on the biological mechanisms underlying the differential response to TNF blocking agents. However, obtaining synovial biopsies is highly invasive and consequently, very few studies have been performed on this key issue with regard to drug efficacy. Using in-house two-color microarrays, van der Pouw Kraan [6] and Lindberg et al. [7] analyzed the baseline transcriptome of RA patients starting infliximab therapy, but neither study found differentially expressed genes. The higher technical variability associated with these early analysis platforms could have undermined the power to accurately identify transcript changes [8]. Using a single-color commercial array, Badot et al. analyzed the longitudinal change in expression of *n* = 8 adalimumab-treated patients [9] and found some expression changes mainly in genes related to cell division. However, differential expression was restricted to 14% of the analyzed transcripts, thereby limiting the global screening capacity to a minor fraction of the total biological variability.

The cellular composition of tissues and the extent of infiltration of immune cell types has proven to be predictive of disease subtypes of complex diseases like cancer [10]. Very recently, studies based on single-cell approaches in the synovial membrane have characterized new pathogenic subsets associated with RA. Using single-cell RNA-seq, Mizoguchi et al. [11] classified RA synovial fibroblasts into three different subsets, with the CD34-THY1+ subtype showing a significant expansion in comparison to osteoarthritis patients. Similarly, using mass-cytometry on T cells isolated from the synovium, Rao et al. [12] found an expanded population of PD-1^hi^CXCR5-CD4+T cells in RA. Due to their capacity to provide B cell help, this new T cell subset was described as the peripheral helper T cell (T_PH_). The relationship of these new pathogenic cell subsets with the response to anti-TNF therapy in RA has not yet been addressed.

In the present study, we have performed a multilevel analysis of the synovial membrane transcriptome to identify critical biological mechanisms associated with response to anti-TNF therapy in RA. Using a longitudinal cohort of patients starting anti-TNF treatment, we identify differentially expressed genes, pathways, and cell types associated with the clinical response at week 20. In the latter analysis, using a cell-deconvolution approach, we find that a lower T_PH_ infiltration is associated with a good response to therapy, and these lower levels are maintained through time. A lower baseline presence of myeloid immune cells is also associated with a favorable response and drastic reduction of this cell compartment is the main biological change observed in responders. The results of this study provide a comprehensive look at the biological mechanisms associated with anti-TNF response in RA.

## Methods

### Patients and samples

Eleven patients (8 women, 3 men) fulfilling the American College of Rheumatology/European League Against Rheumatism 2010 criteria for RA [13], with a basal mean Disease Activity Score in 28 joints (DAS28) [14] of (5.3 [4.2–6.9]) (median and interquartile range [IQR]), non-responder to methotrexate therapy and who were prescribed an anti-TNF by their rheumatologist (*n* = 6 infliximab, *n* = 3 adalimumab, *n* = 2 etanercept), were enrolled in this study. At the time of enrolment, all patients were naïve to any biological therapy. Response to treatment was defined by the EULAR response criteria [15] after 20 weeks of therapy, aggregating moderate and good responders into a single responder group. During the 20-week treatment with anti-TNF, all patients followed the standard treatment pattern, with no adjustments. The study was approved by the local ethics committee, and all patients signed the informed consent. This study was conducted in accordance with the Declaration of Helsinki principles.

Synovial samples were obtained by guided arthroscopy using a 2.7-mm arthroscope (Storz, Tuttlingen, Germany) under local anesthesia. In all patients, 6–8 biopsies were taken from the suprapatellar pouch and medial and lateral gutters with a 3-mm grasping forceps before starting a TNF antagonist. All patients underwent a second arthroscopy with synovial sampling after 20 weeks of therapy. Synovial biopsies for mRNA analysis were immediately stored in the RNALater preserving agent (QIAGEN, USA) and frozen to − 80 °C until RNA extraction [16].

### Microarray analysis

RNA was extracted from synovial biopsies using the RNA Mini Kit (Qiagen, USA), and the integrity was assessed using BioAnalyzer microfluidic gel analysis (Agilent, USA). All samples were of high quality (RNA Integrity Number > 8) and were subsequently analyzed using Sentrix whole genome Beadchips WG6 version 2 (Illumina, US). Briefly, after RNA isolation, biotin-labeled cRNA was prepared using the Ambion Illumina RNA amplification kit (Ambion, US) and Illumina TotalPrep RNA Amplification Kit (Ambion, US). Biotin-labeled cRNA (1.5 μg) was hybridized to WG6 v2 Beadchips and scanned on the 500x Illumina BeadStation. Data collection was performed using BeadStudio 3.1.1.0 software (Illumina, USA).

To perform an independent validation of the cell type associated with treatment response, publicly available transcriptomic datasets (microarray or RNA-seq) were searched in the Gene Expression Omnibus database (https://www.ncbi.nlm.nih.gov/geo/). Using the keywords “synovial membrane” and “rheumatoid arthritis,” a total of 62 datasets were found that were performed on human samples. From these, after excluding in vitro culture studies and two-color microarrays, only two datasets, GSE57376 [17] and GSE15602 [9] on anti-TNF treatment were found. The former dataset was discarded since it was only based on 3 RA patients and had no information on clinical response. The latter dataset consisted of synovial membrane biopsies of *n* = 11 RA patients (3 Good, 5 Moderate and 3 None EULAR responders) were collected at week 12 of adalimumab treatment.

### Gene-level and pathway-level association with anti-TNF response

After log_2_ transformation, raw gene expression data was quantile-normalized. Batch adjustment was performed using ComBat [18]. Differential gene expression was assessed using the *t*-test, and the resulting *P* values corrected for multiple testing using Bonferroni. The biological pathway association was tested using the Gene Ontology (GO) enrichment approach [19] implemented in the *clusterProfiler* R package. Genes with a nominally significant differential expression and a fold change > 1.2 were selected as input for this analysis; Bonferroni multiple test correction was applied to determine significant GO terms.

### RT-QPCR analysis of associated genes

Real-time quantitative PCR analyses of the genes most significantly associated with treatment response and marker genes for PD-1^hi^CXCR5-CD4+ T_PH_ cells were performed using Taqman assays (Life Technologies, USA). In the former group, FAM-labeled assays were used to measure the expression of *PIK3CD* (Hs00192399_m1) and *CX3CL1* (Hs00171086_m1) genes, while *TIMELESS* (Hs01086960_g1) and *CTLA4* (Hs01011591_m1) were used as T_PH_-associated markers. *Ribosomal Protein L11* (VIC-labeled assay, Hs00831112_s1) was used as an endogenous control gene, and all assays were performed using the Taqman Universal PCR master mix (Life Technologies, USA). Gene expression analysis was performed using the recommended protocol with the Applied Biosystems HT7900 system (ABI, USA), and fold-change expression was calculated from the Ct values of the test and endogenous genes using the -2^ΔΔCt^ method. The correlation between microarray gene expression values and RT-PCR values were calculated using Pearson’s product-moment correlation.

### Deconvolution analysis of pathogenic cell subsets

In order to estimate the enrichment of relevant T cell and fibroblast subsets in the synovial membrane samples, we adapted the SPEC (Subset Prediction from Enrichment Correlation) method [20]. Briefly, this computational method uses a gene signature characteristic of specific cell types to estimate their contribution to the observed gene expression in a complex tissue like blood. The ranking of the cell-type signature genes within the global ranked list of genes is then used to compute the enrichment score with an adaptation of the Gene Set Enrichment Method [21]. The enrichment score corresponds to the maximum deviation from zero of a running sum statistic calculated by running down the ranked list of genes. This approach has proven to have a high accuracy in determining cell subsets within PBMC RNA analysis. Here, this deconvolution method was adapted to estimate fibroblast and T cell subtypes from synovial membrane transcriptomic data. To estimate the enrichment of the fibroblast types, we determined the top set of genes overexpressed in each subset. For this objective, we used the microarray gene expression data generated from the three isolated cell types by Mizoguchi et al. [11] and available at the GEO ID GSE107105 (supplementary Table 1). In the case of PD-1^hi^CXCR5-CD+ T cells, we used the set of *n* = 54 significantly overexpressed genes in T_PH_ reported in the original discovery study [12] (supplementary Table 2).

### Immunofluorescence analysis of T_PH_ in RA synovial samples

Immunofluorescence analysis of the T_PH_ lymphocytes was performed following the same methodology as described in the original discovery study [12]. Briefly, OCT frozen sections (7 μm) of the synovial membrane biopsies of the 11 patients treated with anti-TNF therapy and at both time points (baseline and week 20) were fixed with ice-cold acetone. Quenching of endogenous peroxidase was performed using Peroxidase-Blocking Solution (ref: S2023, Dako-Agilent). Blocking of unspecific unions was done using 5% goat normal serum (Life technologies, 16210064) and 2.5% bovine serum albumin (Sigma-Aldrich, 10735078001). Primary antibodies for PD-1 (NAT105,– ref.: 760-4895, Roche), CD4 (SP35, ref: 790-4423, Roche) and CXCR5 (51505, – ref: MAB190, R&D Systems) were used RTU for PD-1 and CD4 and 1:50 for CXCR5 to detect the presence of PD-1^hi^CXCR5-CD4+ cells. As secondary antibodies, Alexa Fluor 488 goat-anti mouse IgG1 (ref: A21121, ThermoFisher), Alexa Fluor Plus 647 goat anti-rabbit IgG (ref: A32733, ThermoFisher), and Biotinilated goat anti-mouse IgG2b (ref: ab98701, Abcam) plus Streptavidin Texas Red® (NEL721001EA, PerkinElmer) were used. Tissue samples were stained with DAPI (D9542, Sigma) and mounted with Fluorescence Mounting Medium (S3023, Dako-Agilent). The specificity of the primary antibodies was determined using rabbit and mouse IgG isotype controls (ref: ab27478 and ab37355, Abcam) and by the omission of the primary antibody.

Digitally scanned fluorescent images were acquired using a NanoZoomer-2.0 HT C9600 scanner (Hamamatsu, Photonics, France) with a ×20 objective and coupled with a mercury lamp unit L11600-05 and using NDP.scan2.5 software U10074-03 (Hamamatsu, Photonics, France). All images were visualized with the gamma correction set at 1.0 and the sharpen filter enabled in the image controls panel of the NDP.view 2 U12388-01 software (Hamamatsu, Photonics, France). Image analysis was performed using QuPath software [22]. Cell detection was performed using the cell detection algorithm based on the DAPI nuclei fluorescence and subsequently, cells were segmented between positive and negative according to the membranous labeling by the three different markers (Alexa 488 for PD-1, Texas Red® for CXCR5 and Alexa 647 for CD4). For each marker, isotype controls as well as negative samples (i.e., samples where the primary antibodies were omitted) were used to set the threshold values for each marker.

## Results

From the 11 RA patients, 8 responded to anti-TNF treatment and 3 patients showed no significant clinical response at week 20 of therapy. The clinical and demographic data of the longitudinal patient cohort are detailed in Table 1.

**Table 1:** Clinical and demographical characteristics of the RA patient cohort

	All ***n*** = 11	EULAR response	
		Good and moderate, ***n*** = 8	No response, ***n*** = 3
Female, *n* (%)	8 (72.7)	5 (62.5)	3 (100)
Age (years)	60.3 (7.1)	60.3 (7.9)	61 (6.9)
Disease duration (years)	17.0 (10.2)	18.4 (10.6)	13.4 (11.3)
Follow-up (years)	2.6 (1.9)	3.6 (1.6)	0.7 (0.8)
DAS28ESR 3v (0 week)	5.3 (1.6)	5.95 (1.4)	5.90 (1.8)
DAS28ESR 3v (20 weeks)	4.2 (1.7)	3.51 (1.3)	5.90 (1.9)
RF positive (%)	6 (54.5)	5 (62.5)	2 (66)
ACPA positive (%)	10 (90.1)	7 (87.5)	3 (100)
**Synovial pattern**			
Lymphoid (%)	7 (63.6)	6 (75)	1 (33.3)
Myeloid (%)	4 (36.3)	2 (25)	2 (66.6)
Fibroid (%)	0	0	0

Clinical and demographical characteristics of the RA patient cohort stratified according to EULAR criteria. None of the variables were significantly different between the two groups (*P* > 0.05)

### Single-gene and pathway association with response

At week 0, *n* = 16 genes were differentially expressed between responders and non-responders (Table 2), from which 11 were overexpressed in responders and 5 in non-responders. At week 20, four genes were differentially expressed after Bonferroni multiple test correction (Table 2), and one of these genes was overexpressed in responders (*PDE4B*, *P* = 1.09E-09) while the remaining 3 were overexpressed in non-responders. At the nominal level, 7 out of the 11 differential genes in the synovial membrane at week 0 continued to be differentially expressed between responders and non-responders at week 20 (*P* < 0.05, the same direction of change, Supplementary Table 3). The remaining 5 genes, while not reaching statistical significance, preserved the same direction of change as found at baseline (supplementary Figure 1). Conversely, none of the four differentially expressed genes at week 20 were differential at week 0 (Supplementary Table 3). This result suggests that the biological features that cause unresponsiveness to TNF blocking therapy prevail throughout anti-TNF treatment. Using RT-PCR, we validated mRNA expression of the gene most differentially expressed at baseline in responders (i.e., *CX3CL1*, correlation *P* value = 2.11e−11) and the gene most highly expressed in non-responders (*PIK3CD*, correlation *P* value = 2.42e−10) (Supplementary Figure 2).

**Table 2:** List of genes differentially expressed between anti-TNF responders and non-responders

Gene symbol	Accession	Definition	Fold change	*P* value
Genes significantly overexpressed in non-responders, week 0				
PIK3CD*	NM_005026.2	Phosphoinositide-3-kinase, catalytic,	− 1.56	7.11e−18
		delta polypeptide		
*REEP4*	NM_025232.2	Receptor accessory protein 4	− 1.50	7.14e−17
*HCLS1*	NM_005335.3	Hematopoietic cell-specific Lyn substrate 1	− 1.63	4.38e−09
*GCDH*	NM_013976.2	Glutaryl-coenzyme A dehydrogenase	− 1.50	3.2e−07
*ADA*	NM_000022.2	Adenosine deaminase	− 1.73	7.15e−07
*NT5DC2*	NM_022908.1	5′-Nucleotidase domain containing 2	− 1.86	7.7e−07
*SERPINH1*	NM_001235.2	Serpin peptidase inhibitor, clade H, member 1	− 1.66	1.67e−06
Genes significantly overexpressed in responders, week 0				
CX3CL1*	NM_002996.3	Chemokine (C-X3-C motif) ligand 1	1.94	7.4e−12
*PLS3*	NM_005032.3	Plastin 3 (T isoform)	1.71	1.97e−10
*DIXDC1*	NM_033425.1	DIX domain containing 1	1.90	9.29e−10
*TMOD1*	NM_003275.1	Tropomodulin 1	2.10	1.56e−08
*PCOLCE2*	NM_013363.2	Procollagen C-endopeptidase enhancer 2	2.45	3.34e−07
*PPP1R3C*	NM_005398.3	Protein phosphatase 1, regulatory (inhibitor)	2.13	3.64e−07
		Subunit 3C		
*CRTAP*	NM_006371.3	Cartilage associated protein	1.58	4.78e−07
*PRELP*	NM_002725.3	Proline/arginine-rich end leucine-rich repeat	2.02	6.42e−07
		Protein, transcript variant 1		
*DKFZP686A01247*	NM_014988.1	Hypothetical protein	2.30	7.13e−07
*UNQ689*	NM_212557.1	RSTI689	5.09	9.54e−07
*OR2A9P*	NR_002157.1	Olfactory receptor, family 2, subfamily A,	2.01	1.94e−06
		member 9		
Genes significantly overexpressed in non-responders, week 20				
*RPS6KA2*	NM_001006932.1	Ribosomal protein S6 kinase, 90 kDa	− 1.53	3.10e−07
		Polypeptide 2		
*LIPA*	NM_000235.2	Lipase A, lysosomal acid, cholesterol esterase	− 1.50	1.50e−07
		(Wolman disease)		
*ALPL*	NM_000478.2	Alkaline phosphatase, liver/bone/kidney	− 2.77	7.90e−07
Genes significantly overexpressed in responders, week 20				
*PDE4B*	NM_002600.2	Phosphodiesterase 4B, cAMP-specific	1.57	1.10e−07

The list of genes that are significant after Bonferroni correction and that show an > 1.5 absolute fold change between the two groups are shown. Fold change is calculated contrasting the mean expression in the anti-TNF responder group to the mean expression in the non-responder. *Top differentially expressed genes validated using RT-QPCR

At the pathway level, at week, 0 *n* = 16 GO terms were significantly enriched in responders, and *n* = 70 GO terms were enriched in non-responders (Table 3 and Supplementary Table 4). At week 20, *n* = 17 GO terms were significantly enriched, and all due to genes overexpressed in anti-TNF non-responders (Table 3 and Supplementary Table 5). From these, three gene ontologies were significantly enriched at baseline in non-responders: GO:0043299|*leukocyte degranulation* (adjusted *P*_wk0_ = 9.8e−4, adjusted *P*_wk20_ = 3.97e−11), GO:0002275|*myeloid cell activation involved in immune response* (adjusted *P*_wk0_ = 3.1e−4, adjusted *P*_wk20_ = 3.6e−10) and GO:0002444|*myeloid leukocyte mediated immunity* (adjusted *P*_wk0_ = 6.98e−3, adjusted *P*_wk20_ = 6.36e−10). This result suggests that the innate immune system composition in the synovial membrane conditions the response to anti-TNF therapy, with non-responders bearing a higher infiltrate of myeloid lineage cells (i.e., neutrophils and macrophages). Comparing the longitudinal changes in biological pathways in the responder group, we found a profound modification with *n* = 149 GO terms significantly enriched from week 0 to week 20 (Table 3 and Supplementary Table 6). Most of the changes are due to the inactivation of pathways (*n* = 123 GO terms, 82.6%), but also several biological processes are activated due to anti-TNF efficacy (*n* = 26 GO terms, 17.4%). In the former group, we found that the biological changes most associated in responders corresponds to the same three GO terms that are consistently different when comparing responders vs non-responders at the two time points: GO:0043299|*leukocyte degranulation* (adjusted *P* = 3.26e−32), GO:0002275|*myeloid cell activation involved in immune response* (adjusted *P* = 1.14e−31) and GO:0002444|*myeloid leukocyte mediated immunity* (adjusted *P* = 2.99e−30). This result corroborates that TNF efficacy is mainly mediated by the drastic inactivation of the innate immune system. Of relevance, the pathways that become activated due to TNF response include biological processes strongly related to the production of the specific components of the joint stromal tissue, including GO:0001501|*skeletal system development* (adjusted *P* = 2.11e−10), GO:0001503|*ossification* (adjusted *P* = 4.04e−8), GO:0001649|*osteoblast differentiation* (adjusted *P* = 2.61e−4), GO:0051216|*cartilage development* (adjusted *P* = 8−42e−4) and GO:0002063|*chondrocyte development* (adjusted *P* = 8.99e−4). This biological evidence shows that the effective clearance of inflammation is paralleled by the activation of joint tissue reconstruction programs.

**Table 3:** Top associated Gene Ontologies with the anti-TNF response at baseline and week 20 and with the longitudinal change

***GOs associated with differential expression: week 0***				
ID	Description	Count	***P*** value	Gene group
GO:0044772	Mitotic cell cycle phase transition	75	3.64e−17	Non-response
GO:0044770	Cell cycle phase transition	77	1.07e−16	Non-response
GO:0140014	Mitotic nuclear division	47	2.39e−13	Non-response
GO:0000280	Nuclear division	56	3.70e−12	Non-response
GO:0051301	Cell division	70	1.81e−11	Non-response
GO:0007346	Regulation of mitotic cell cycle	71	2.04e−11	Non-response
GO:0048285	Organelle fission	57	4.90e−11	Non-response
GO:0000070	Mitotic sister chromatid segregation	31	5.86e−11	Non-response
GO:0000819	Sister chromatid segregation	33	3.24e−10	Non-response
***GOs associated with differential expression: week 20***				
GO:0043299	Leukocyte degranulation	40	3.97e−11	Non-response
GO:0042119	Neutrophil activation	38	1.07e−10	Non-response
GO:0036230	Granulocyte activation	38	1.46e−10	Non-response
GO:0002275	Myeloid cell activation involved in immune response	39	3.61e−10	Non-response
GO:0002444	Myeloid leukocyte mediated immunity	39	6.36e−10	Non-response
***GOs associated with longitudinal change in responders***				
**Gene Ontology**	**Description**	**Count**	***P*** **value**	**Gene group**
GO:0043299	Leukocyte degranulation	74	3.24e−32	Downregulated
GO:0002275	Myeloid cell activation involved in immune response	74	1.14e−31	Downregulated
GO:0002444	Myeloid leukocyte mediated immunity	73	2.99e−30	Downregulated
GO:0036230	Granulocyte activation	68	1.40e−28	Downregulated
GO:0042119	Neutrophil activation	67	5.88e−28	Downregulated
GO:0002283	Neutrophil activation involved in immune response	65	1.18e−26	Downregulated
GO:0002446	Neutrophil mediated immunity	65	5.17e−26	Downregulated
GO:0043312	Neutrophil degranulation	64	5.65e−26	Downregulated
GO:0030198	Extracellular matrix organization	53	4.20e−16	Upregulated
GO:0043062	Extracellular structure organization	53	4.01e−13	Upregulated
GO:0009617	Response to bacterium	50	1.64e−11	Downregulated
GO:0002764	Immune response-regulating signaling pathway	45	2.69e−11	Downregulated
GO:0002757	Immune response-activating signal transduction	42	2.06e−10	Downregulated
GO:0002250	Adaptive immune response	39	2.23e−10	Downregulated
GO:0045088	Regulation of innate immune response	37	6.63e−10	Downregulated
GO:0001501	Skeletal system development	54	7.19e−10	Upregulated

List of the most significantly associated (*P* < 1e−9, multiple test adjusted) Gene Ontologies in the three pathway level analyses based on three analyses: (i) genes differentially expressed in the synovial membrane between anti-TNF responders and non-responders at baseline, (ii) genes differentially expressed between anti-TNF responders and non-responders at week 20, and (iii) genes differentially expressed between week 0 and week 20 in the anti-TNF responder group

*ID* Gene Ontology ID, *Count* number of differentially expressed genes that belong to the GO, *P value* significance of the observed enrichment (Bonferroni-corrected for the number of total GOs tested), *Gene Group* differential expression group from which the genes were selected to test for enrichment

### Cell type deconvolution of RA pathological cell subtypes

**Fig. 1:**
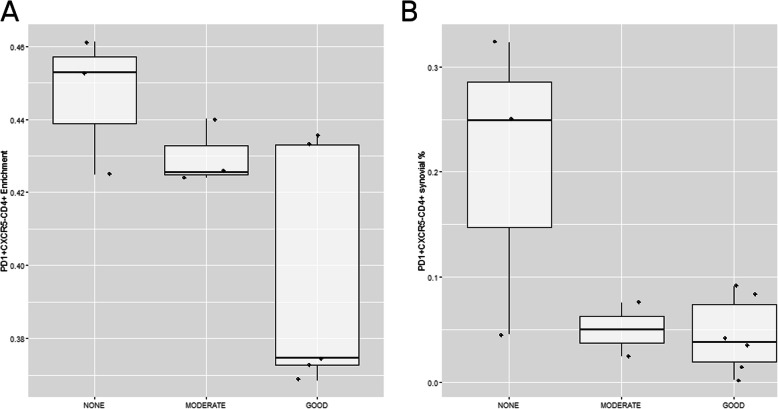
The T_PH_ enrichment in the synovial membrane of RA patients at baseline therapy is associated with the clinical response. **a** PD-1^hi^CXCR5-CD4+ cell enrichment levels at baseline estimated using the cell-type deconvolution approach in our patient cohort. The estimated enrichment scores for T_PH_ cells are significantly associated with the EULAR response at week 20 (*P* = 0.021). **b** PD-1^hi^CXCR5-CD4+ cell percentage at baseline quantified using immunofluorescence in our patient cohort. As seen in the cell-type deconvolution approach, lower T_PH_ cells are associated with better responses to anti-TNF agents (*P* = 0.025). In both cases, moderate and good EULAR responders show distinctively lower proportions of this pathogenic T cell subset associated with RA

Using single-cell characterization technologies, recent studies have identified distinct cell subsets within RA synovium. In particular, two new subtypes peripheral helper CD4+ T cells and THY1+ synovial fibroblasts have been found to be key pathological types. Based on the genes that are the characteristic of each subtype and using a deconvolution approach, we estimated the relative proportion of each cell type. We found at baseline, response to anti-TNF therapy was associated with having lower numbers of T_PH_ cells (*P* = 0.021, Fig. 1a). We did not find evidence that THY1+ fibroblasts were associated with the response to anti-TNF therapy. Instead, we observed that the baseline proportions of both CD34+THY1- and CD34-THY1- (i.e., double negative) fibroblasts were associated with a response at week 20 (*P* = 0.027 and *P* = 0.021, respectively, Supplementary Figure 3). At the time of response (week 20), we found that all three associated cell types (CD34+ and CD34-THY1- fibroblasts and T_PH_) showed the same trend of association as identified at baseline, although the difference was not statistically significant (*P* > 0.05, supplementary Figure 4).

To perform technical validation of the observed gene expression associated with T_PH_ cells, we performed RT-PCR analysis of T_PH_-marker genes *CTLA4* and *TIMELESS*. We found a highly significant correlation between the microarray and the RT-PCR gene expression quantification (Pearson correlation coefficients *r* = 0.89, *P* = 3.6e−8 and *r* = 0.65 *P* = 0.0009 for *CTLA4* and *TIMELESS*, respectively) (Supplementary Figure 5).

### Immunofluorescence analysis of T_PH_ cells

**Fig. 2:**
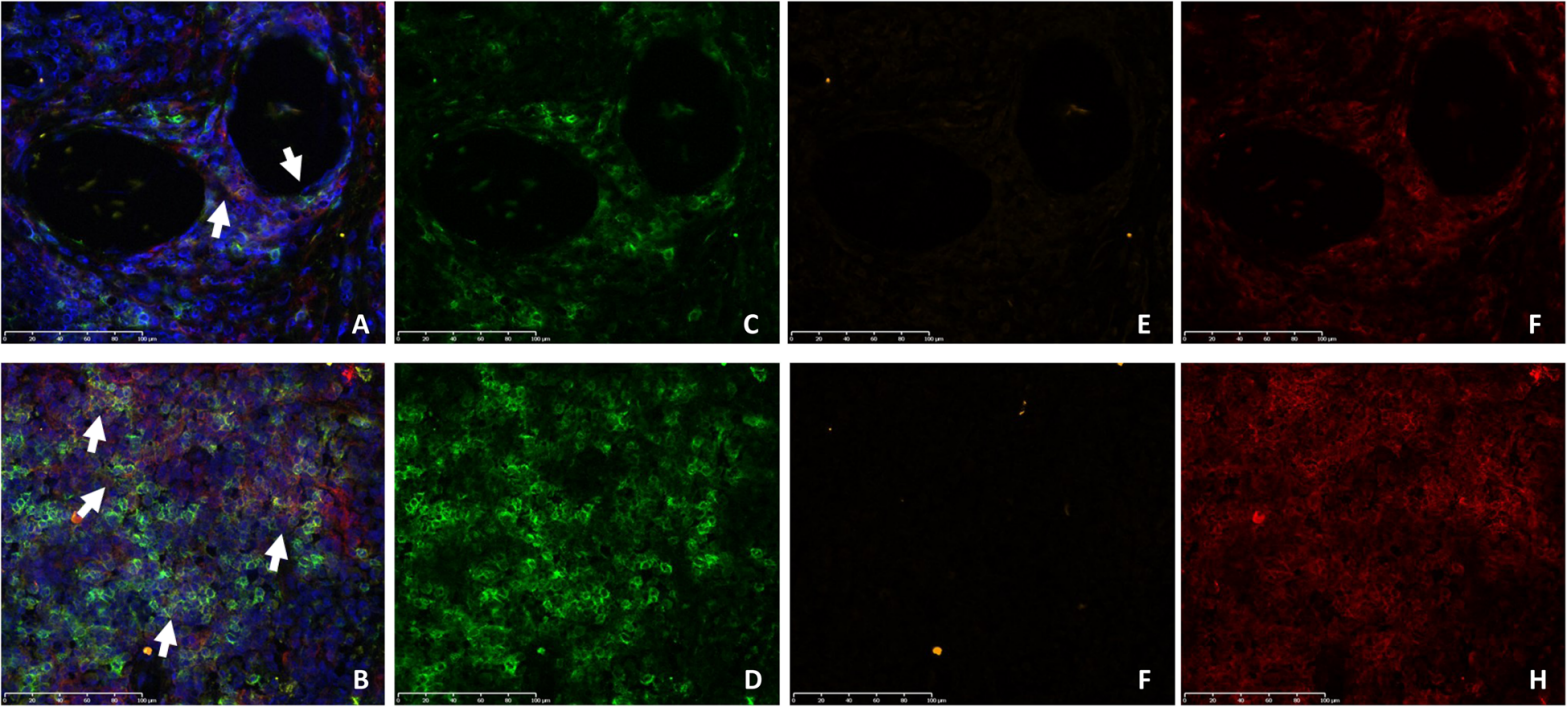
Representative immunofluorescence of T_PH_ in synovial tissues of a good responder (**a**, **c**, **e**, **g**) and a non-responder (**b**, **d**, **f**, **h**) at week 20. **c**, **d** PD1-positive cells in green, **e**, **f** CXCR5-positive cells in orange, and **g**, **h** CD4-positive cells in red. **a** and **b** show the combined immunofluorescence image of a good and bad responder to anti-TNF therapy, respectively. White arrows indicate synovial membrane aggregations of CD4+PD1+ T cells

In order to corroborate the association between T_PH_ enrichment and anti-TNF response, we performed immunofluorescence analysis on the synovial membrane biopsies from the same set of patients (Fig. 2). All samples were positive for all markers which presented a membranous pattern. From them, CXCR5 expression was minimal in comparison with PD-1 and CD4. Anti-TNF non-responder patients presented higher groups of PD-1^hi^CXCR5-CD4^+^ T cells (Fig. 1b, *P* = 0.025). We found a significant correlation between the proportions of PD-1^hi^CXCR5-CD4+ T cells estimated through our approach and the actual abundance of this pathogenic T cell subtype in the RA synovia (*r*^2^ = 0.58, *P* = 0.0051, Supplementary Figure 6).

**Fig. 3:**
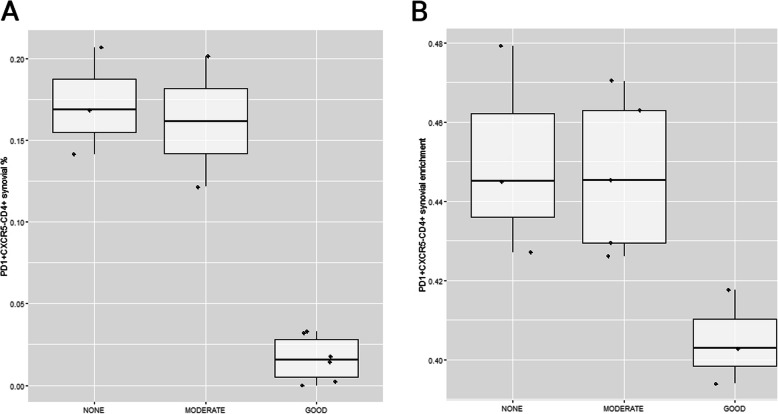
T_PH_ enrichment analysis in the synovial membrane of the external dataset reproduces the patterns observed in our patient cohort. **a** PD-1^hi^CXCR5-CD4+ cell percentage quantified using immunofluorescence on synovial biopsies of our patient cohort at week 20 of anti-TNF therapy. **b** PD-1^hi^CXCR5-CD4+ cell enrichment levels estimated using the cell-type deconvolution approach on the external patient cohort at week 12 of anti-TNF therapy. In both cases, EULAR good responders show distinctively lower T_PH_ cell levels than moderate or none responders (*P* = 3.5e−4 and *P* = 4e−3 for our patient cohort and the external RA patient cohort, respectively)

Having confirmed the association of T_PH_ cells with the response to anti-TNF therapy, we applied the cell-type deconvolution method to an external microarray dataset of RA patients treated with anti-TNF therapy [9]. In the transcriptional data at week 12 of therapy (*n* = 3 Good, 5 Moderate, and 3 None), we found a highly similar T_PH_ profile to that of patients at week 20 (*P* = 0.029 and *P* = 0.00019, respectively) (Fig. 3). In both cases, good responders show a markedly lower frequency of the pathogenic T cell subset (*P* = 0.00035 and *P* = 0.0040, *t* test comparing T_PH_ cell levels in good vs moderate and none patients in our patient cohort and the external RA patient cohort, respectively).

## Discussion

The reasons behind the variable efficacy of anti-TNF agents in RA have been elusive. Using single-gene, pathway and cellular deconvolution analyses on longitudinal transcriptomic data from the synovial membrane of RA patients treated with anti-TNF therapy, we have found key biological determinants of the response to this drug. At the gene level, baseline overexpression of the chemokine *CX3CL1* and downregulation of leukocyte signaling kinase *PIK3CD* were significantly associated with a better response. At the pathway level, anti-TNF non-responders had a higher activation of myeloid cell immune response processes at baseline. A drastic downregulation of these same innate immunological processes was strongly associated with the efficacy of the drug at week 20. At the cellular level, lower T_PH_ cells were associated with a good response to anti-TNF agents, both at baseline and at week 20. The results from this study provide a multilevel description of the biological determinants of anti-TNF response in RA.

Recent studies based on single-cell analysis techniques have characterized new pathogenic cell subtypes associated with RA. Using single-cell mass cytometry, PD-1^hi^CXCR5-CD4+ T (T_PH_) cells were found to be significantly expanded in the synovial membrane of seropositive RA patients. Using the genes characterizing this T cell subset, we were able to estimate the presence of a significantly lower number of T_PH_ cells in responders to anti-TNF therapy. This difference was clearly maintained up to week 20 in the good responder group of patients and validated in an independent cohort of patients. The transcriptional similarity between PD-1^hi^CXCR5-CD4+ T and follicular helper T (T_FH_) and the in vitro capacity to induce differentiation of memory B cells suggests a peripheral helper phenotype. Our results suggest that an important part of this T_PH_ pathogenic activity in RA is associated with mechanisms that are independent of TNF. T_PH_ may act by recruiting T_FH_ and B cells, promoting local autoantibody production that may not be reflected in the serum, leading to pathogenic immune complex fixation. Furthermore, activated B cells can polarize into different subtypes to produce an array of proinflammatory cytokines other than TNF [23], including interferon-gamma, IL-6, and IL-17. The latter cytokine, for example, promotes the accumulation of neutrophils in the synovial fluid, where they produce large amounts of cartilage-degrading enzymes and reactive oxygen species [24]. This way, in patients with higher levels of T_PH_, the inflammation will have alternative mechanisms to progress despite the reduction of TNF levels by anti-TNF agents.

Our results also suggest that the stromal component of the synovial membrane participates in the favorable response to anti-TNF therapy. Single-cell analysis of the synovial membrane identified three different synovial fibroblast types [11]: CD34-THY1-, CD34-THY1+, and CD34+. CD34-THY1+ fibroblasts have been shown to be expanded when compared with osteoarthritis synovium and therefore have been identified as the principal pathogenic subset. In our cell type analysis, we did not find evidence for enrichment of CD34-THY1+ fibroblasts with anti-TNF response. Instead, we observed a significant increase of CD34+ and CD34-THY1- fibroblasts. As described previously [25], THY1 is also highly abundant in vascular cell types, and therefore, an immunofluorescence-based quantification of this fibroblast subtype was ruled out. Therefore, these results should be taken with caution. Nonetheless, functional evidence indicates that CD34+ fibroblasts have a leading role in the recruitment of monocytes into the inflamed synovial, producing large amounts of IL6, CXCL2, and CCL2 in response to TNF stimulation. In comparison, CD34-THY1-fibroblasts produce higher levels of matrix metalloproteinases MMP1 and MMP3. According to our results, both fibroblast types would cause more TNF-sensitive stromal cells, in which a reduction in local levels of this cytokine by therapy would be translated into a less proinflammatory and matrix-degrading activity and, ultimately, into a higher likelihood of clinical response. Recently, Croft et al. [25] have further refined the classification of synovial fibroblasts in RA by identifying fibroblast activation protein α (FAPα+) as a key stratification marker. Like in the previous study, THY1-fibroblasts were shown to localize in the synovial lining layer and specifically correlate with cartilage-damaging features. Instead, FAPα+ THY1+ fibroblasts were shown to correlate with markers of systemic and tissue inflammation. Studies integrating this refined fibroblast characterization into the association with the response to anti-TNF therapy in RA are therefore warranted.

At the single gene level, we found two genes, *CX3CL1* and *PIK3CD*, showing distinctively high expression levels at baseline in responders and non-responders, respectively. From these, only *CXC3L1* remains overexpressed in the synovial membrane of responders at week 20. *CXC3L1*, also known as fractalkine, is a chemokine that can be present as membrane-bound or in soluble forms and is highly expressed in endothelial cells activated by TNF [26]. Interaction of membrane-bound fractalkine with its receptor CX3CR1 mediates the capture of circulating immune cells and infiltration into the inflamed tissue. In particular, CX3CR1^hi^ monocytes that patrol endothelium have been shown to rapidly accumulate in sites after activation of the endothelium [27]. Accordingly, our results are indicative of patients with vascular tissue richer in CX3CL1 expression are more likely to have a beneficial impact of TNF blocking.

*PIK3CD* is highly expressed in the synovial membrane of patients that will not respond to therapy. Together with *PIK3CA* and *PIK3CB*, *PIK3CD* is a class I phosphoinositide 3-kinase, which is a group of enzymes that phosphorylate inositol lipids and participates in different signaling processes [28]. While *PIK3CA* and *PIK3CB* are expressed in many different tissues, *PIK3CD* is limited to the cells of the immune system including T and B lymphocytes [29], neutrophils [30], and macrophages [31]. More recently, *PIK3CD* has also been found to be highly expressed in RA synovial fibroblasts [32]. In vitro studies have shown that TNF is a major inducer of *PIK3CD* expression in synovial fibroblasts but not in leukocytes [33]. In these previous studies, the expression of *PIK3CD* in RA synovial fibroblasts was shown to induce cell growth by sensitizing fibroblasts to platelet-derived growth factor (PDGF) [32]. Based on this evidence, non-responders would have a stromal component more prone to PDGF-derived hyperplasia. For this group of patients, a combination of drugs interfering with PDGF signaling might be an efficacious approach to rescue non-responder patients. Experimental and clinical evidence of the therapeutic potential of PDGF pathway interference in RA is clearly supported [34].

The pathway-level analysis of anti-TNF therapy response revealed an important contribution of the myeloid cell load. Our longitudinal data demonstrates that a high myeloid cell activity not only conditions for a poorer response, but also, that it is the biological process which is more highly altered by the drug’s efficacy. The existence of two patient groups in RA with different myeloid leukocyte activation is in high accordance with recent findings using a single-cell RNA-seq analysis. Analyzing the synovial membrane cell composition of a relatively large patient cohort of RA and osteoarthritis individuals, Zhang et al. [35] identified two subtypes of patients in RA, defined as leukocyte-poor RA and leukocyte-rich RA. Our study extends these findings into the therapeutic domain. Despite not showing distinguishable clinical activities at baseline, non-responder patients have a significantly higher myeloid activity, which is one of the top-ranking biological processes detected in the leukocyte-rich RA patients. It is important to note that, using a standard histological definition of synovial tissue (i.e., lymphoid/myeloid/fibroid), we found no association with a clinical response or with T_PH_ proportions (data not shown). This suggests that more precise, molecular quantification techniques like transcriptomic analysis are needed to capture this patient variability. In this regard, the pathway analysis of genes associated with baseline with the response to anti-TNF therapy is in line with previous transcriptomic evidence [9], in which poor responders showed a predominance of pathways associated with cell cycle regulation. This common observation indicates that in those patients where there is very active cell division in the synovial membrane, the TNF blockade will likely be inefficient. Our analysis did not allow us to differentiate however if this higher cell cycle activation is due to the immune or stromal cell compartments (or both). Further exploring this biological feature could provide a simple means to classify RA patients that will likely be resistant to the therapeutic effect of anti-TNF agents.

While our results show an association between a new pathogenic cell type and the response to therapy, this does not imply that this information is sufficient to be used to predict treatment response in RA. Aspects like effect size and technical and biological variation can hinder the capacity of a biological marker like T_PH_ to predict anti-TNF response. Examples of the disparity between association and prediction have been shown previously for example in genetic polymorphisms associated with the disease risk with insufficient disease prediction [36]. To fill this gap, specific studies will need to be carried out to evaluate the predictor performance of this new biomarker in extended patient cohorts under different technologies and varying clinical features. In this regard, finding less invasive biomarkers like blood analytes that correlate T_PH_ abundance would therefore be a useful means to simplify patient recruitment and stratification and test its utility to predict response to anti-TNF therapy.

## Conclusions

In the present study, we have performed a multilevel analysis of the RA synovial membrane transcriptome in relation to the anti-TNF therapy response. Using a cell-type deconvolution approach, we have found that a newly characterized pathogenic T cell subtype in RA, peripheral T helper cell, is associated with the lack of response to TNF blocking. Our longitudinal analysis shows that good response at week 20 is preserved by a sustained low T_PH_ cell number. At the pathway level, response to anti-TNF treatment is strongly linked to an efficacious clearance of myeloid leukocyte activation. At the single-gene level, two genes previously associated with RA pathology, *CX3CL1* and *PIK3CD*, were found to be markers for the anti-TNF response, implicating variability in the immune cell recruitment and fibroblastic growth capacities of the synovial membrane. The results from this study indicate new biological features of anti-TNF response in RA and could be useful starting points to allow for patient stratification and for the development of new therapeutic approaches, alone or in combination with TNF blockers, to achieve a favorable response for a larger number of patients.

## Supplementary information

**Additional file 1 : Supplementary Table 1**. Marker genes of the three synovial membrane fibroblast types. **Supplementary Table 2.** Significantly overexpressed genes in PD-1^hi^ T cells according to Rao et al. **Supplementary Table 3.** Preservation of gene expression differences between baseline and response time point (week 20). **Supplementary Figure 1. Preservation of differential expression changes associated to anti-TNF response in the synovium from baseline. Supplementary Figure 2**. RTPCR replication of *CX3CL1* and *PIK3CD* genes. **Supplementary Figure 3**. Association of the three fibroblast subsets at baseline with anti-TNF response. **Supplementary Figure 4**. Cell types associated with anti-TNF response: differences at week 20. **Supplementary Figure 5**. QRTPCR replication of T_PH_ biomarker genes *CTLA4* and *TIMELESS*. **Supplementary Figure 6**. Correlation between PD1^hi^CXCR5-CD4+ T cell abundance estimation using the cell deconvolution approach with the percentage quantified using immunofluorescence.

**Additional file 2 :: Supplementary Table 4.** Significant GO terms of genes differentially expressed at week 0 between responders and non-responders to anti-TNF therapy. **Supplementary Table 5.** Significant GO terms of genes differentially expressed at week 20 between responders and non-responders to anti-TNF therapy. **Supplementary Table 6.** Significant GO terms of genes differentially expressed between week 0 and week 20 in the responder group of patients.

## Data Availability

The datasets generated and analyzed during the current study are available at the NCBI Gene Expression Omnibus database (https://www.ncbi.nlm.nih.gov/gds) with accession numbers GSE140036, GSE47726, and GSE15602.

## Abbreviations

EULAR: European Leagues Against Rheumatism
GO: Gene ontology
mRNA: Messenger RNA
RA: Rheumatoid arthritis
RT-PCR: Real-time polymerase chain reaction
TNF: Tumor necrosis factor-alpha
T_PH_: Peripheral helper T cells
