# Supplementary material for: Lower peripheral helper T cell levels in the synovium are associated with a better response to anti-TNF therapy in rheumatoid arthritis
Source: Arthritis Res Ther. 2020 Aug 25;22:196. doi: 10.1186/s13075-020-02287-9 (PMC7446220; doi:10.1186/s13075-020-02287-9)
Supplement: Supplementary file 1 — Additional file 1 : Supplementary Table 1. Marker genes of the three synovial membrane fibroblast types. Supplementary Table 2. Significantly overexpressed genes in PD-1hi T cells according to Rao et al. Supplementary Table 3. Preservation of gene expression differences between baseline and response time point (week 20). Supplementary Figure 1. Preservation of differential expression changes associated to anti-TNF response in the synovium from baseline. Supplementary Figure 2. RTPCR replication of CX3CL1 and PIK3CD genes. Supplementary Figure 3. Association of the three fibroblast subsets at baseline with anti-TNF response. Supplementary Figure 4. Cell types associated with anti-TNF response: differences at week 20. Supplementary Figure 5. QRTPCR replication of TPH biomarker genes CTLA4 and TIMELESS. Supplementary Figure 6. Correlation between PD1hiCXCR5-CD4+ T cell abundance estimation using the cell deconvolution approach with the percentage quantified using immunofluorescence. [file 13075_2020_2287_MOESM1_ESM.docx]

**Supplementary Material**

Table of Contents

[Supplementary Table 1. Marker genes of the three synovial membrane fibroblast types. 2](#_Toc45888199)

[Supplementary Table 2 3](#_Toc45888200)

[Supplementary Table 3. Preservation of gene expression differences between baseline and response time point (week 20). 4](#_Toc45888201)

[Supplementary Figure 1. Preservation of differential expression changes associated to anti-TNF response in the synovium from baseline. 6](#_Toc45888202)

[Supplementary Figure 2. RTPCR replication of CX3CL1 and PIK3CD genes. 7](#_Toc45888203)

[Supplementary Figure 3. Association of the three fibroblast subsets at baseline with anti-TNF response. 8](#_Toc45888204)

[Supplementary Figure 4. Cell types associated with anti-TNF response: differences at week 20. 11](#_Toc45888205)

[Supplementary Figure 5. QRTPCR replication of T_PH_ biomarker genes CTLA4 and TIMELESS. 14](#_Toc45888206)

[Supplementary Figure 6. Correlation between PD1^hi^CXCR5-CD4+ T cell abundance estimation using the cell deconvolution approach with the percentage quantified using immunofluorescence. 15](#_Toc45888207)

# Supplementary Table 1. Marker genes of the three synovial membrane fibroblast types.

**CD34+ markers**

| *ADAM22* | *CACHD1* | *FZD4* | *PCDH18* |
| --- | --- | --- | --- |
| *ADAMTSL3* | *CAMK2D* | *G0S2* | *PKNOX2* |
| *AHR* | *CD34* | *GDA* | *RHOU* |
| *ANGPT1* | *CEP126* | *IQCC* | *SETBP1* |
| *ASPA* | *CLIP3* | *LPCAT2* | *SSPN* |
| *BICC1* | *CLLU1OS* | *MAB21L2* | *TBX5* |
| *C3* | *CP* | *MECOM* | *TMEM108* |
| *C6* | *CXCL12* | *NOVA1* | *TMEM133* |
| *C7* | *EGR1* | *OSR2* | *TRDMT1* |

**THY1+ markers**

| *ADCY7* | *CCNG1* | *FLRT2* | *PLXDC1* | *THY1* |
| --- | --- | --- | --- | --- |
| *ADGRA2* | *COMP* | *FNDC1* | *PRKD1* | *TTC39C* |
| *ANKRD29* | *CRABP2* | *IGF1* | *PTGIS* | *VSTM4* |
| *ARL4C* | *CRISPLD2* | *LTBP2* | *SFRP4* | *WISP1* |
| *ARSB* | *CUTC* | *MBNL2* | *SMOC2* |  |
| *ASPN* | *DPP4* | *MCUB* | *SRGAP1* |  |
| *AXIN2* | *EDIL3* | *MYOF* | *STXBP5* |  |
| *C12orf75* | *ELN* | *NLGN2* | *THBS1* |  |
| *CADM1* | *FHL2* | *OAF* | *THBS2* |  |

**CD34-THY1- markers**

| *ADAM10* | *ERRFI1* | *MET* | *PPIL6* | *TIMP3* |
| --- | --- | --- | --- | --- |
| *ADGRA3* | *EZR* | *MFSD6* | *RBPMS2* | *TMCO3* |
| *ADGRG2* | *FNIP2* | *MSN* | *RELL1* | *TMEM196* |
| *AGFG1* | *FOXO1* | *MT1G* | *RGS16* | *TSPAN15* |
| *BTC* | *GALNT12* | *MTHFD1L* | *RIOK3* | *TWISTNB* |
| *C10orf105* | *GFPT2* | *MTUS2* | *SEC11C* | *UBAP1* |
| *CLIP1* | *GPR18* | *NDP* | *SHTN1* | *UGP2* |
| *CNTNAP3* | *GPR183* | *NTN4* | *SIX3* | *USP24* |
| *CSN1S1* | *GPX3* | *OSTF1* | *SORBS2* | *UST* |
| *CUX1* | *GRAMD1B* | *PACSIN2* | *SOX5* | *VPS13A* |
| *CXCL8* | *HBEGF* | *PCSK6* | *STK38L* | *WARS* |
| *DAPK1* | *HMGA2* | *PDE8A* | *SUSD4* | *ZNF385B* |
| *DNASE1L3* | *HTRA4* | *PDLIM5* | *SV2B* |  |
| *ENPP1* | *KIF1B* | *PGM3* | *SYT17* |  |
| *EREG* | *LRRFIP2* | *PNP* | *TIAM2* |  |

Genes differentially overexpressed from each fibroblast subset compared to the other two were selected as markers for cell type deconvolution analysis.

Supplementary Table 2**. Significantly overexpressed genes in PD-1^hi^ T cells according to Rao et al.**

| *PD-1* | *GZMK* | *F5* |
| --- | --- | --- |
| *SCO2* | *DHFR* | *DUSP2* |
| *CDCA7* | *TIMELESS* | *MAF* |
| *TOX* | *DUSP4* | *PSMA4* |
| *TOX2* | *FABP5* | *SYT11* |
| *ENC1* | *FBXO41* | *UBE2A* |
| *HVCN1* | *MAP3K9* | *TBC1D4* |
| *CHN1* | *PMAIP1* | *UQCRC1* |
| *CCDC86* | *PRR5L* | *ANXA2* |
| *CCL5* | *MIS18BP1* | *SLAMF6* |
| *AKR1C3* | *EPSTI1* | *TUBB4B* |
| *CST7* | *BZRAP1* | *UBE2L6* |
| *CEP128* | *DPP3* | *ITM2A* |
| *MYL6B* | *TIGIT* | *RGS1* |
| *EZH2* | *FCRL3* |  |
| *CXCR3* | *CTLA4* |  |
| *ICA1* | *DDX54* |  |
| *FANCI* | *RAB37* |  |
| *FAM210A* | *SHMT2* |  |
| *DIRC2* | *PFN1* |  |

# Supplementary Table 3. Preservation of gene expression differences between baseline and response time point (week 20).

| **Symbol** | **Accession** | **Definition** | **FCh wk0** | **P wk0** | **FCh wk20** | **P wk20** |
| --- | --- | --- | --- | --- | --- | --- |
| ***Significant genes at baseline*** | | |  |  |  |  |
| *PIK3CD* | NM_005026.2 | phosphoinositide-3-kinase, catalytic, delta polypeptide (PIK3CD) | -1.56 | 7.10E-18 | -1.22 | 0.3 |
| *CX3CL1* | NM_002996.3 | chemokine (C-X3-C motif) ligand 1 (CX3CL1) | 1.94 | 7.40E-12 | 1.96 | **0.0019** |
| *PLS3* | NM_005032.3 | plastin 3 (T isoform) (PLS3) | 1.71 | 2.00E-10 | 1.65 | 0.059 |
| *DIXDC1* | NM_033425.1 | DIX domain containing 1 (DIXDC1) | 1.90 | 9.30E-10 | 1.69 | **0.01** |
| *HCLS1* | NM_005335.3 | hematopoietic cell-specific Lyn substrate 1 (HCLS1) | -1.63 | 4.40E-09 | -1.68 | 0.076 |
| *TMOD1* | NM_003275.1 | tropomodulin 1 (TMOD1) | 2.10 | 1.60E-08 | 1.76 | **0.029** |
| *PCOLCE2* | NM_013363.2 | procollagen C-endopeptidase enhancer 2 (PCOLCE2) | 2.45 | 3.30E-07 | 2.39 | 0.061 |
| *PPP1R3C* | NM_005398.3 | protein phosphatase 1, regulatory (inhibitor) subunit 3C (PPP1R3C) | 2.13 | 3.60E-07 | 2.09 | **0.011** |
| *CRTAP* | NM_006371.3 | cartilage associated protein (CRTAP) | 1.58 | 4.80E-07 | 1.33 | 0.11 |
| *PRELP* | NM_002725.3 | proline/arginine-rich end leucine-rich repeat protein (PRELP), transcript variant 1 | 2.02 | 6.40E-07 | 1.79 | 0.11 |
| *DKFZP686-A01247* | NM_014988.1 | hypothetical protein (DKFZP686A01247) | 2.30 | 7.10E-07 | 1.78 | **0.033** |
| *ADA* | NM_000022.2 | adenosine deaminase (ADA) | -1.73 | 7.10E-07 | -1.45 | 0.08 |
| *NT5DC2* | NM_022908.1 | 5'-nucleotidase domain containing 2 (NT5DC2) | -1.86 | 7.70E-07 | -1.23 | 0.44 |
| *UNQ689* | NM_212557.1 | RSTI689 (UNQ689) | 5.09 | 9.50E-07 | 5.53 | **0.0018** |
| *SERPINH1* | NM_001235.2 | serpin peptidase inhibitor, clade H (heat shock protein 47), member 1, (collagen binding protein 1) (SERPINH1) | -1.66 | 1.70E-06 | -1.30 | 0.5 |
| *OR2A9P* | NR_002157.1 | olfactory receptor, family 2, subfamily A, member 9 pseudogene (OR2A9P) on chromosome 7. | 2.01 | 1.90E-06 | 1.71 | **0.0047** |
| ***Significant genes at week 20*** | | |  |  |  |  |
| *RPS6KA2* | NM_001006932.1 | ribosomal protein S6 kinase, 90kDa, polypeptide 2 | -1.58 | 0.074 | -1.53 | 3.10E-07 |
| *LIPA* | NM_000235.2 | lipase A, lysosomal acid, cholesterol esterase (Wolman disease) | -1.14 | 0.34 | -1.50 | 1.50E-07 |
| *ALPL* | NM_000478.2 | alkaline phosphatase, liver/bone/kidney | -1.38 | 0.58 | -2.77 | 7.90E-07 |
| *PDE4B* | NM_002600.2 | phosphodiesterase 4B, cAMP-specific | 1.03 | 0.88 | 1.57 | 1.10E-07 |

List of genes that are significant after Bonferroni correction and with an (absolute) >1.5 fold-change (FC) between responders and non-responders to anti-TNF therapy at week 0 and week of response. In bold, the genes at week 20 that preserve the differential expression between the two groups at the nominal level (P < 0.05). Conversely, none of the differentially expressed genes at week 20 had a nominally significant difference at baseline.


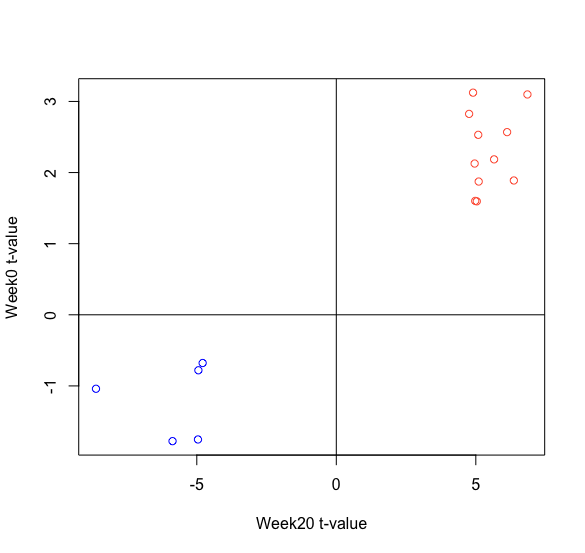


Supplementary Figure 1. Preservation of differential expression changes associated to anti-TNF response in the synovium from baseline. The t-values of the genes that are differential at week 0 between responders and non-responders (n=16) are plotted against the t-values of the same genes at week 0. While not all differences are significant at week 20, it the direction of the change is clearly preserved in all overexpressed (red, n=11) and underexpressed (blue, n=5) genes.

# Supplementary Figure 2. RTPCR replication of *CX3CL1* and *PIK3CD* genes.

The gene expression of the two genes more differentially expressed at baseline between responders and non-responders was validated using Taqman QRTPCR assays. The correlation between both platform was highly significant (r=0.86 P=2.42e-10 and r=0.90 P=2-11e-11 for PIK3CD and CX3CL1, respectively).

# Supplementary Figure 3. Association of the three fibroblast subsets at baseline with anti-TNF response.

**
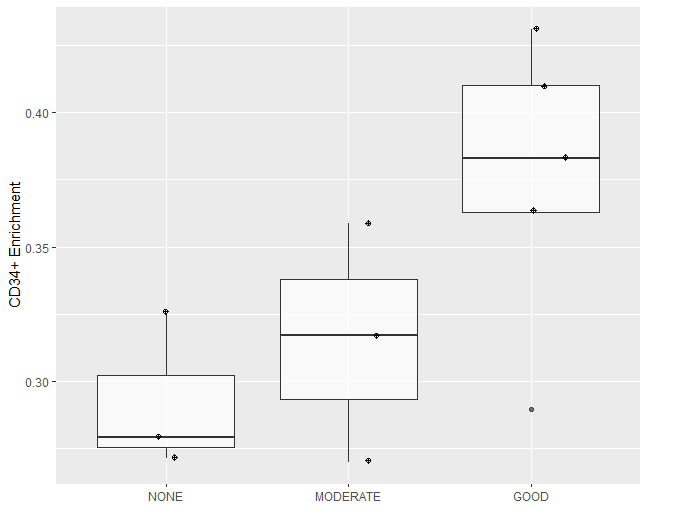
**

1. ***CD34+ fibroblast enrichment in the synovial membrane in our patient cohort at baseline according to the response to anti-TNF therapy.*** Improvement in response increases linearly with the proportion of CD34+ fibroblast in the synovial membrane (P=0.027).


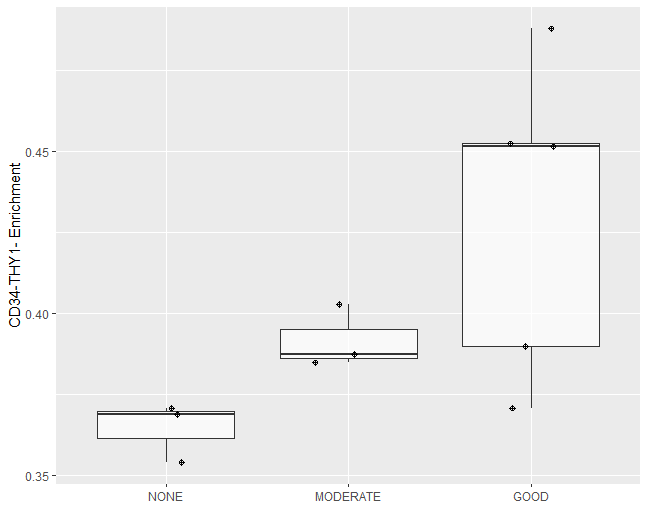


1. ***CD34-THY1- fibroblast enrichment in the synovial membrane in our patient cohort at baseline according to the response to anti-TNF therapy.*** Improvement in response increases linearly with the proportion of CD34-THY1- fibroblasts in the synovial membrane (P=0.021).


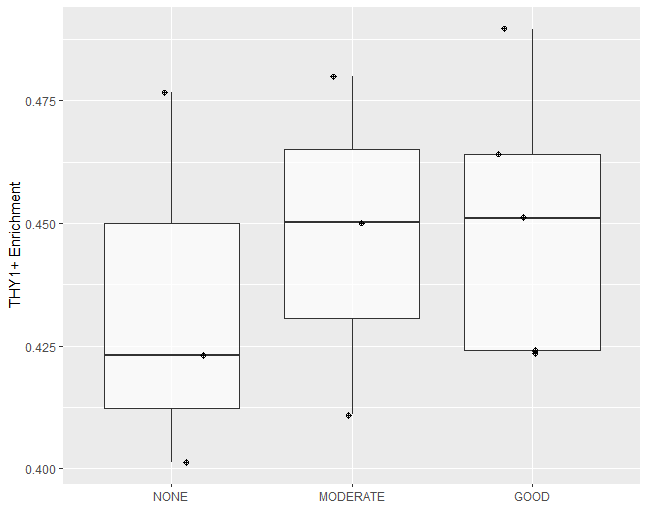


1. ***Boxplot of THY1+ fibroblast enrichment in the synovial membrane in our patient cohort at baseline according to the response to anti-TNF therapy.*** The estimated proportion of THY1+ fibroblasts is not significantly associated with the response to anti-TNF therapy (P=0.49).

# Supplementary Figure 4. Cell types associated with anti-TNF response: differences at week 20.

**
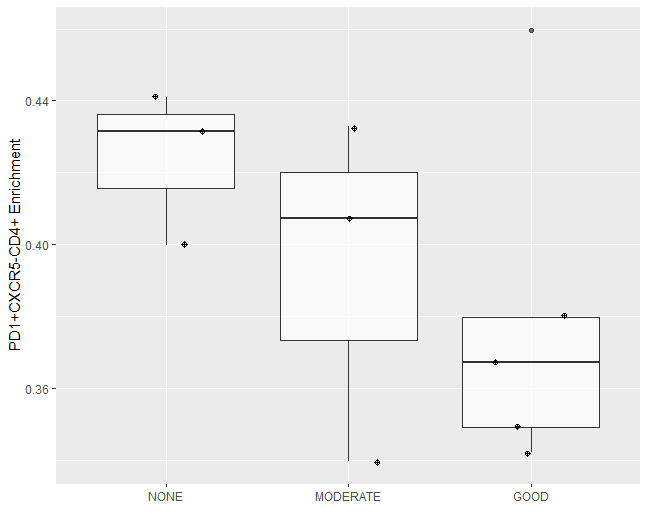
**

1. ***Boxplot of PD1^hi^CXCR5-CD4+ T cell* *enrichment in the synovial membrane in our patient cohort at week20 according to the response to anti-TNF therapy.*** The trend between lower T_PH_ cells and a better response to anti-TNF therapy identified at baseline is maintained up to week 20 of therapy. Despite this, the association is no longer significant (P=0.29).


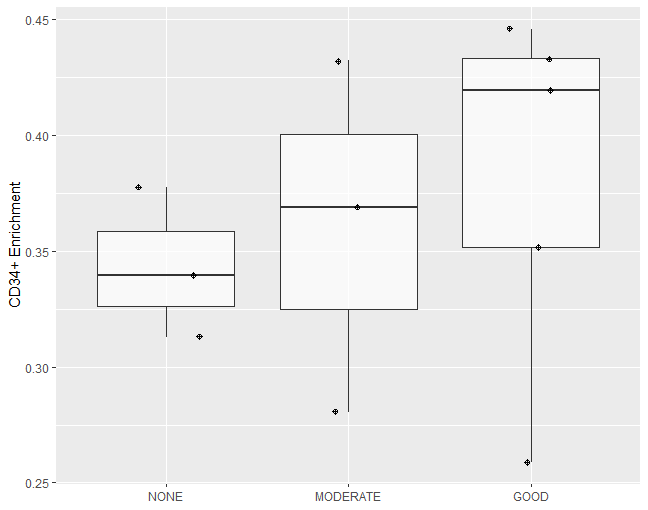


1. ***Boxplot of CD34+ fibroblast* *enrichment in the synovial membrane in our patient cohort at week 20 according to the response to anti-TNF therapy.*** The trend between higher CD34+ fibroblasts and a better response to anti-TNF therapy identified at baseline is maintained up to week 20 of therapy but is no longer significant (P=0.43).


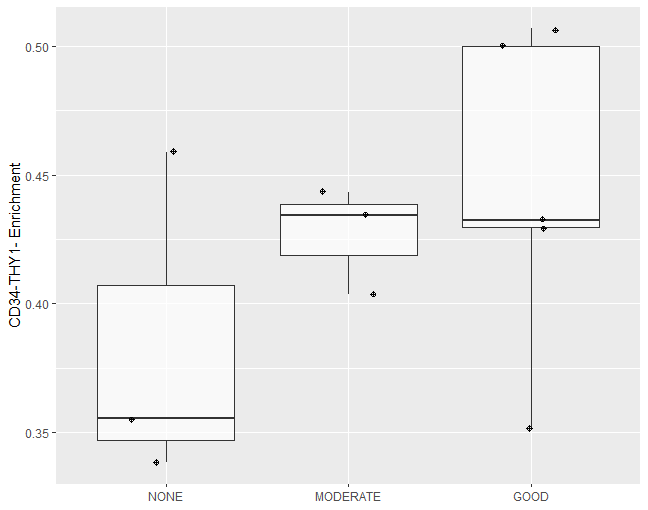


1. ***Boxplot of CD34-THY1- fibroblast* *enrichment in the synovial membrane in our patient cohort at week 20 according to the response to anti-TNF therapy.*** The trend between higher *CD34-THY1-* fibroblasts and a better response to anti-TNF therapy identified at baseline is maintained up to week 20 of therapy but is no longer significant (P=0.16).

# Supplementary Figure 5. QRTPCR replication of T_PH_ biomarker genes *CTLA4* and *TIMELESS*.

Plot of gene expression measures for *CTLA4* and *TIMELESS* as quantified using Illumina Beadchip microarrays and using Taqman quantitative RTPCR. The high concordance between both platforms confirms the validity of the microarray gene expression quantification for accurate cell type deconvolution estimation.

# Supplementary Figure 6. Correlation between PD1^hi^CXCR5-CD4+ T cell abundance estimation using the cell deconvolution approach with the percentage quantified using immunofluorescence.


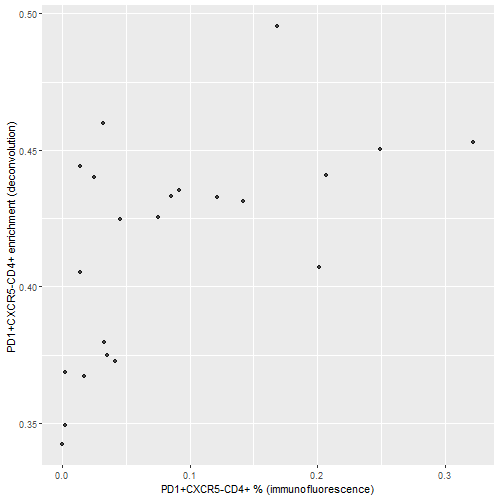


The estimated proportion of T_PH_ cells in the synovial membrane using the cell-type deconvolution approach shows a high correlation with the percentage of cells directly quantified using immunofluorescence on the synovial membrane samples (r2=0.58, P=0.0051). T_PH_ values for all n=11 for the two time points (baseline and wk20) are plotted. This result confirms the suitability of cell type deconvolution approach to estimate even low frequency cell types like T_PH_.
